# The Relationship Between Neutrophil to Lymphocyte Ratio and Clinical Outcome in Pediatric Patients After Cardiopulmonary Bypass Surgery: A Retrospective Study

**DOI:** 10.3389/fped.2019.00308

**Published:** 2019-07-31

**Authors:** Hao Xu, Yanxin Sun, Sibi Zhang

**Affiliations:** Department of Anesthesiology, The First Affiliated Hospital of Nanjing Medical University, Nanjing, China

**Keywords:** pediatric patients, cardiopulmonary bypass, neutrophil lymphocyte ratio, C reactive protein, prognostic

## Abstract

**Background:** The aim of this study was to investigate the prognostic role of the neutrophil to lymphocyte ratio (NLR) in pediatric patients undergoing open-heart surgery with cardiopulmonary bypass (CPB).

**Methods:** A retrospective cohort study of 61 pediatric patients who underwent CPB in the First Affiliated Hospital of Nanjing Medical University from January 2017 to October 2018 was conducted. All perioperative clinical data, including neutrophil count, lymphocyte count, and C-reactive protein (CRP), were collected retrospectively. The prognostic value of NLR and its association with extubation time, duration of intensive care unit (ICU) stay and in-hospital stay were analyzed.

**Results:** Neutrophil count, NLR, and CRP were significantly increased post-operative compared with pre-operative (*p* < 0.05). The increased post-operative levels of NLR were significantly associated with longer extubation time, as well as prolonged post-operative cardiac ICU stay (*p* < 0.05). Multivariate linear regression analysis revealed that elevated NLR displayed a significant independent association with extubation time and the duration of an ICU stay.

**Conclusion:** Increased post-operative NLR followed by cardiac surgery with CPB in pediatric patients was associated with longer extubation time and a longer duration of an ICU stay.

## Introduction

Previous studies have demonstrated that a systemic inflammatory response is activated in patients undergoing cardiothoracic surgery with cardiopulmonary bypass (CBP) (1, 2). Multiple studies have demonstrated a relationship between altered production of inflammatory mediators and clinical outcome in pediatric patients after CPB (3, 4). However, few inflammatory biomarkers have been used for outcome evaluation in the clinical practice. Therefore, the identification and quantification of clinical factors in the early post-operative period could be predictive of increased risk of post-operative morbidity and poor outcomes is imperative.

Neutrophil activation can trigger systemic inflammation and has been recognized to play a pivotal role in the inflammatory response induced by CPB (5, 6). Moreover, the neutrophil to lymphocyte ratio (NLR) is a novel inflammatory marker which has been found to be associated with the severity and prognosis of many cardiovascular diseases (7). Importantly, increased perioperative NLR was shown to be associated with poor cardiac surgery patient outcomes in adult patients (8, 9). Despite this, NLR has not been well-studied in exclusively pediatric patients undergoing cardiac surgery requiring CPB. Therefore, the objective of the present study is to examine the association between NLR levels and clinical outcomes in pediatric patients undergoing cardiac surgery with CPB.

## Methods

### Study Design, Variables, and Patients

This is a retrospective observational study. From January 2017 to October 2018, a total of 61 pediatric patients undergoing cardiac surgery requiring CPB were included. This study was approved by the hospital's institutional review board (IRB). Since no intervention was performed and no personally identifiable information was used in the study, our local IRB waived the requirement for informed consent. The inclusion criteria of this study were: patients were to be aged between 3 and 72 months and underwent pediatric cardiac surgery requiring CPB. Exclusion criteria for this study included a birth weight of <2.3 kg, associated extracardiac abnormalities, and a history of cardiac surgery.

### Anesthetic and CPB Managements

Anesthesia and management of CPB were performed using standard techniques depending on the age, weight, and hematocrit (HCT) level of the pediatric patients. Briefly, anesthesia was induced with 8% sevoflurane, followed by the placing of a peripheral venous catheter. After an intravenous injection of fentanyl (5 μg/kg), cisatracurium (0.15 mg/kg), propofol (3 mg/kg), and midazolam (0.05 mg/kg), endotracheal intubation was performed. Pressure-controlled ventilation was performed, and the frequency of respiration was adjusted based on the patient's age as well as partial pressure of arterial carbon dioxide. Propofol (8 mg/kg/h), cisatracurium (2 μg/kg/min), and sevoflurane (1–3%) were used for the maintenance of anesthesia. Fentanyl (20–30 μg/kg) and midazolam (0.1 mg/kg) were added for the maintenance of anesthesia. All subjects received methylprednisolone (30 mg/kg) and cefazolin sodium (50 mg/kg) before skin incision. None received intraoperative steroids.

The CPB circuit was primed with acetated Ringer's solution, red blood cells (RBCs), plasma, 20% mannitol, 5% sodium bicarbonate, and heparin. HCT was maintained at 24–28% during CPB. Heparin was given at a dose of 3 mg/kg to achieve a target activated clotting time of 480 s. After the initiation of CPB, moderate hypothermia was started with a target rectal temperature of 28 to 32°C. The pump flow rates ranged from 2 to 3 L/min/m^2^. The mean arterial blood pressure during CPB was maintained between 35 and 60 mmHg according to the patient's age. Dopamine (5 μg/kg/min) and milrinone (0.5 μg/kg/min) were routinely used to facilitate weaning off from CPB. Ultrafiltration was used during and/or after termination of CPB. Platelets and cryoprecipitate were regularly used after CPB. RBCs and fresh frozen plasma were given at the anesthesiologist's discretion. The reversal of heparin after termination of bypass was achieved with the administration of protamine (3 mg/kg).

### Data Collection

All data was collected retrospectively in November 2018. Pre-operative and post-operative levels (first post-operative day) of neutrophils, lymphocytes, and CRP were obtained through the electronic database of the hospital. NLR was calculated on the basis of data. Clinical patient data were collected through medical and nursing patient record review.

### Statistical Methods

Categorical and numerical data are expressed as *n* and mean ± SD, respectively. Post-operative neutrophil counts, NLR and CRP were compared with pre-operative counts by using the paired *t*-test. Spearman rank correlation coefficients were used to assess the association between neutrophil counts, NLR, and CRP and continuous clinical outcomes. Multivariable linear regression was used to further examine the relationship between post-operative neutrophil counts, NLR, and CRP and clinical outcome variables, including extubation time, the duration of an ICU stay, and length of hospital stay. The significance level was defined as a *p* < 0.05. All statistical analyses were performed using the statistical software SPSS 21.0 (SPSS Inc., Chicago, IL).

## Results

During study period (January 2017 to October 2018), 61 children (*n* = 39 male, *n* = 22 female) were included in the final analysis. Diagnoses of 61 patients included congenital heart defect ventricular septal defect (*n* = 28), atrial septal defect (*n* = 2), ventricular septal defect with atrial septal defect (*n* = 7), tetralogy of Fallot (*n* = 5), tetralogy of Fallot with atrial septal defect (*n* = 4), complete endocardial cushion defect (*n* = 3), pulmonary atresia (*n* = 4), and complex congenital heart disease (*n* = 9).

The median age was 20.4 ± 19.2 months (ranging from 3 to 69.5 months). The mean duration of ICU and in-hospital stay were 1.705 ± 0.955 days (ranging from 1 to 5 days) and 10.393±2.795 days (ranging from 7 to 17 days), respectively. The mean time of extubation was 16.346 ± 19.539 h (ranging from 1.2 to 92.8 h). A detailed table of the subjects' baseline and operative characteristics in comparison with the entire cohort is presented in Table 1.

**Table 1:** Baseline characteristics and clinical parameters.

**Variable**	**Value**
Male/Female	39/22
Age at surgery (months)	20.4 ± 19.2 (3–69.5)
Weight (kg)	10.889 ± 6.925 (5–41)
Height (cm)	78.721 ± 18.85 (50–150)
BMI	16.518 ± 3.135 (11.45–28)
Diagnoses	
VSD	28
ASD	2
ASD/VSD	7
TOF	5
TOF/ASD	4
CECD	3
PA	4
CCHD	9
Cardiopulmonary bypass time (minutes)	87.213 ± 34.00 (36–179)
Cross-clamp time (minutes)	57.672 ± 28.018 (17–141)
Operation time(minutes)	208.180 ± 53.203 (110–362)
Extubation time	16.346 ± 19.539 (1.2–92.8)
ICU stay	1.705 ± 0.955 (1–5)
Length of hospital stay	10.393 ± 2.795 (7.0–17.0)
Pre-operative neutrophil	3.318 ± 1.524
Post-operative neutrophil	10.864 ± 3.926
Pre-operative lymphocyte	5.881 ± 2.775
Post-operative lymphocyte	1.659 ± 0.811
Pre-operative NLR	0.718 ± 0.562
Post-operative NLR	8.166 ± 5.539

*VSD, ventricular septal defect; ASD, atrial septal defect; VSD/ASD, ventricular septal defect with atrial septal defect; TOF, tetralogy of Fallot; TOF/ASD, tetralogy of Fallot with atrial septal defect; CECD, complete endocardial cushion defect; PA, pulmonary atresia; CCHD, complex congenital heart disease*.

**Figure 1:**
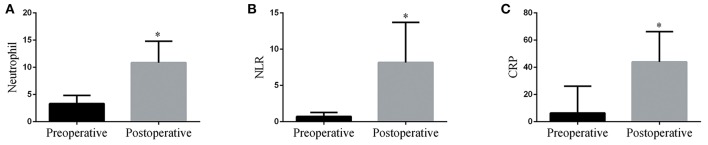
Post-operative neutrophil counts **(A)**, NLR **(B)**, and CRP **(C)** levels were significantly increased compared with their respective pre-operative values in pediatric patients following CPB. ^*^p < 0.05 vs. pre-operative.

The data showed that post-operative neutrophil counts, as well as CRP and NLR levels were significantly higher compared to their respective pre-operative levels in pediatric patients requiring CPB (Figure 1, *p* < 0.05). Using a univariate analysis of clinical outcomes (Table 2), it was revealed that the post-operative NLR was significantly associated with the duration of surgery, extubation time, and length of ICU stay (*p* < 0.05). However, both pre-operative and post-operative neutrophil levels were significantly associated with length of ICU stay and extubation time (*p* < 0.05). In addition, the post-operative CRP levels were associated with CPB duration, circulatory arrest duration and length of ICU stay (*p* < 0.05).

**Table 2:** Spearman rank correlation coefficients of neutrophil count, NLR, and CRP levels measured before and after operation with clinical outcome.

	**Pre-operative**			**Post-operative**		
	**Neutrophil count**	**NLR**	**CRP**	**Neutrophil count**	**NLR**	**CRP**
Body Mass Index	0.375	0.214	0.624	0.375	0.791	0.344
Cardiopulmonary bypass time	–	–	–	0.687	0.31	0.022
Circulatory arrest time	–	–	–	0.597	0.335	0.041
Operation time	–	–	–	0.529	**0.037**	0.375
Extubation time	0.028	0.140	0.067	**0.028**	**0.021**	0.453
ICU stay	0.014	**0.034**	0.191	**0.014**	**0.001**	**0.012**
Length of hospital stay	0.560	0.503	0.883	0.560	0.450	0.513

*Bold values indicate p < 0.05*.

We then set out to determine whether post-operative NLR maintained an independent association with extubation time, duration of ICU stay, and length of hospital stay when controlling for key operative factors, as well as post-operative neutrophil counts and CRP levels. Multivariable modeling results showed that post-operative NLR demonstrated an independent association with extubation time and length of ICU stay (Table 3, *p* < 0.05).

**Table 3:** Multivariate analysis for duration of ICU stay, extubation time and length of hospital stay.

	**ICU stay**		**Extubation time**		**Length of hospital stay**	
	**Coefficient**	***p*-value**	**Coefficient**	***p*-value**	**Coefficient**	***p*-value**
Cardiopulmonary bypass time	0.390	0.254	1.433	0.158	−0.885	0.380
Circulatory arrest time	0.090	0.753	0.397	0.693	1.945	0.057
Intraoperative red blood cells	0.020	0.882	−0.497	0.621	−0.319	0.751
Intraoperative plasma	−0.056	0.690	0.368	0.714	0.889	0.378
Operation time	−0.239	0.208	−1.514	0.136	1.065	0.292
Post-operative neutrophil	0.093	0.529	0.722	0.474	0.941	0.351
Post-operative NLR levels	−0.350	**0.027**	−2.129	**0.038**	−1.010	0.317
Post-operative CRP levels	−0.113	0.376	0.485	0.629	−0.272	0.787

*Bold values indicate p < 0.05*.

## Discussion

The main findings of this study were that CRP, NLR, and neutrophil counts were significantly increased post-operatively compared with pre-operatively. In addition, the post-operative neutrophil counts and NLR on the first post-operative day after surgery were significantly correlated with extubation time and duration of ICU stay. However, the post-operative CRP was only associated with ICU stay. After adjustment for key operative factors, post-operative neutrophil counts, and CRP levels, the elevated NLR on the first post-operative day was independently associated with extubation time and length of ICU stay. Our data suggests that the NLR could be an adjunct indicator for post-operative outcome in pediatric patients who had undergone CPB.

Previous research has demonstrated that CPB-induced systemic inflammatory response is a major cause of mortality (10, 11). Neutrophils are the common type of leukocytes, and arrive rapidly to sites of acute inflammation, where they are responsible for phagocytosis and killing of invading pathogens (12). Neutrophil activation is known to trigger systemic inflammation and has been demonstrated to play a pivotal role in the inflammatory response induced by CPB (6, 13, 14). CPB triggers the activation of neutrophils in the complement cascade, inducing the secretion of polymorphonuclear elastase (PMN-E). Overactivation of PMN-E on one hand can directly induce cell injury and, on the other hand, indirectly enhances the inflammatory response by promoting the synthesis and release of IL-8 through the IL-1 signaling (15). Previous research has demonstrated a positive correlation between plasma concentrations of IL-8 at the end of CPB with duration of CPB and circulatory arrest, as well as with aortic cross-clamp time, and a negative correlation with lowest temperature on CPB (3). The pre-treatment with the neutrophil elastase inhibitor sivelestat could suppress the perioperative inflammatory response following pediatric cardiac surgery with CPB (15, 16). Our current data demonstrate that a significant increase of neutrophil levels is associated with increased post-operative extubation time and increased length of ICU stay. Taken together with findings from previous research studies, our results demonstrate the need for targeted anti-inflammatory therapy in the pediatric population, with a specific focus on the neutrophil cells.

In adult patients, an augmentation in perioperative NLR was shown to be associated with increased mortality and morbidity in patients undergoing cardiac surgery (8). In comparison with adult patients, the inflammatory response is increased in pediatric patients undergoing CPB (1). Many potential prognostic and predictive molecular inflammatory biomarkers have now been identified in the evaluation of poor outcomes following CPB; however, none have yet entered into clinical practice. Accumulating evidence demonstrated that NLR could reflect a systemic inflammatory response (17, 18); however, there is only one study evaluating the prognosis associations of NLR in pediatric patients with CPB (19). According to the results of the study, the NLR measured before extubation displayed significantly more variation in pediatric patients with failed extubation; less variation in NLR was found in children in whom extubation was successful (20). In addition, pre-extubation NLR was indicative of successful extubation (20). In the current study, we also found that post-operative NLR was significantly increased in pediatric patients compared to pre-operative NLR. In addition, there was a significant and positive correlation between the duration of ventilation and serum NLR in pediatric patients after cardiac surgery with CPB. Lastly, our data showed that post-operative NLR was also associated with a longer duration of post-operative ICU stay. These findings suggest that monitoring of the NLR has the potential to make predictions about probable outcomes in pediatric patients undergoing cardiac surgery with CPB.

C-reactive protein (CRP) is an acute-phase pentameric protein secreted in response to inflammation (20). Previous data showed that serum CRP levels significantly increased after CPB compared with pre-operative levels (21). The current data presents both NLR and CRP levels in the same pediatric patients requiring CPB. Despite significantly increased post-operative CRP levels compared with pre-operative levels, there was no significant association between post-operative CRP levels, extubation time, and length of ICU stay after adjusting for post-operative neutrophil counts, NLR, and key operative factors. These results suggest that NLR would be more effective than CRP to monitor patients with delayed tracheal extubation and predict length of ICU stay in pediatric patients requiring CPB.

The present study has some limitations. Firstly, the study was conducted in only one hospital. Therefore, a multicenter study needs to be carried out in order to further contribute to our findings. Secondly, due to the retrospective design of our study, it is potentially susceptible to systematic error and bias. However, the numerical data used in our study were collected prospectively and analyzed independently. Thirdly, our data examination was limited to the period which ended upon patients' discharge from the hospital, and further research is needed to examine the long-term impact of NLR on patient outcomes. Finally, the current study examined a fairly wide age group which covers a diverse group of underlying congenital heart defects, and future research will further develop and confirm these initial findings by narrowing the age range for each type of heart disease.

In conclusion, elevated post-operative NLR was associated with increased extubation time and an increased length of ICU stay for pediatric patients after CPB surgery. As NLR can be easily calculated using the complete blood count, it can potentially provide a simple and cost-effective test in the management of pediatric cardiac surgery patients.

## Data Availability

The raw data supporting the conclusions of this manuscript will be made available by the authors, without undue reservation, to any qualified researcher.

## Ethics Statement

This study was approved by the institutional review board (IRB) of The First Affiliated Hospital With Nanjing Medical University. Since no personally identifiable information was used in the study, our local IRB waived the requirement for informed consent.

## Author Contributions

HX and SZ designed the study, interpreted results, and wrote the manuscript. YS collected data.

### Conflict of Interest Statement

The authors declare that the research was conducted in the absence of any commercial or financial relationships that could be construed as a potential conflict of interest.
